# A crowded podium in hemophilia A: when a low annualized bleeding rate is no longer enough to tell the contenders apart

**DOI:** 10.1016/j.rpth.2026.106942

**Published:** 2026-09-04

**Authors:** Laurent Frenzel

**Affiliations:** 1 Haematology Unit and Haemophilia Treatment Centre, Necker-Enfants Malades Hospital and Cochin Hospital, AP-HP, Imagine Institute, Paris, France; 2 Benign Hematology Unit, Memorial Sloan Kettering Cancer Center, New York, New York, USA

## 1 Introduction

For most of its modern history, the story of hemophilia A has been told through a single number: the annualized bleeding rate (ABR). From on-demand treatment to standard, and then extended half-life factor VIII (FVIII) concentrates, and from the first bispecific antibody to today’s rebalancing agents, each therapeutic generation has been asked, first and foremost, to lower the ABR. It is therefore all the more striking to read, in the FRONTIER2 extension study reported by Jiménez-Yuste et al. [1], that denecimig (Mim8), a next-generation, fully human activated FVIII mimetic, maintained mean ABRs of treated bleeds between 0.21 and 2.02 across treatment arms over 52 weeks, with median ABRs of 0 in nearly every group, and the majority of baseline target joints resolved. These are, by any classical standard, excellent results. They extend to a full year of sustained protection, first demonstrated in the FRONTIER2 26-week main phase, recently published in the New England Journal of Medicine, where denecimig reduced the ABR by 96%-99% when compared to prior on-demand treatment and by 43%-54% when compared to prior clotting factor concentrate prophylaxis [2].

And yet, precisely because the results are so good, they raise a question the authors could not have anticipated when the FRONTIER program was designed: if almost every new molecule now drives ABR close to 0, how are clinicians, regulators, and payers to tell the contenders apart? The hemophilia pipeline has become genuinely crowded, but the classical yardstick we have always used to choose between them is losing its power to discriminate.

## 2 Efficacy: Remarkable Results that are Increasingly Hard to Rank

The efficacy of denecimig in FRONTIER2 is genuinely impressive, especially in the prestudy on-demand group switched to prophylaxis, where the 52-week estimated mean ABR fell to 0.37 (once-weekly) and 0.21 (once-monthly) [1]. An antibody administered subcutaneously as infrequently as once-a-month, delivering this degree of protection irrespective of inhibitor status, would have been almost unimaginable a decade ago. The mechanistic basis is a molecule engineered for high cofactor activity, with roughly 13- to 18-fold greater potency than a sequence-identical analog of emicizumab in thrombin-generation and clot-formation assays [3], translating into robust clinical efficacy at low, fixed subcutaneous doses [4,5].

The difficulty is one of comparison. Placed alongside the most recent phase 3 datasets, denecimig’s numbers are excellent but no longer uniquely so (Table). Long-term pooled data from the HAVEN 1–4 studies show that emicizumab maintains a model-based treated ABR of 1.4, falling below 1 over time, with roughly 82% of patients bleed-free in the 121 to 144 week interval [6]. Efanesoctocog alfa, the first “ultra-long” FVIII, produced a mean ABR of 0.71 in XTEND-1 while restoring near-normal FVIII activity for much of the week [7]. The rebalancing agents cluster in a similar range: fitusiran under its revised antithrombin-based regimen achieved a median ABR of 3.7 with as few as 6 injections per year [8]; concizumab and marstacimab reduced bleeding by roughly 86% and 92%, respectively, compared with no prophylaxis in their pivotal trials [[9], [10], [11]]. In other words, denecimig joins a field in which every serious candidate already delivers a low single-digit, often near-zero, ABR.

**Table 1:** Selected phase 3 and extension-study results for novel therapies relevant to haemophilia A

Therapy (class)	Trial / follow-up	Treated ABR Mean (median)	Zero treated bleeds, %	Selected safety findings	Route / interval
**Denecimig / Mim8 (FVIIIa mimetic)**	FRONTIER2 52 weeks^a^	0.21–2.02^a^	49–88%^a^	No TE; no clinical evidence of neutralising ADA; ISRs <2% of injections	SC, weekly or monthly
**Emicizumab (FVIIIa mimetic)**	HAVEN 1–4 pooled Median 120 weeks	1.4	82.4% (weeks 121–144)	TMA/TE associated with high-dose aPCC^b^	SC, every 1, 2 or 4 weeks
**Efanesoctocog alfa (ultra-long FVIII)**	XTEND-1 52 weeks	0.71 (0)	64.7% (52 weeks)	No FVIII inhibitors detected	IV, weekly
**Fitusiran (AT-lowering siRNA)**	ATLAS-OLE AT-based regimen	6.4 (3.7)	31.5% (primary efficacy period)	Thrombotic events; ALT elevations	SC, monthly or every 2 months^c^
**Concizumab (anti-TFPI)**	explorer7 Primary analysis^d^	1.7 (0)	63.6% (first 24 weeks)	TE prompted pause; revised dosing at restart	SC, daily
**Marstacimab (anti-TFPI)**	BASIS 12 months^e^	3.2^e^	—	No TE in the reported trial period	SC, weekly
**Valoctocogene roxaparvovec (gene therapy)**	GENEr8-1 Year 5	0.6 (0)	77.8% (year 5)	ALT elevations	One-time IV infusion^f^

Results are descriptive, not head-to-head comparisons. Populations, inhibitor status and assessment periods differ; ATLAS-OLE, explorer7 and BASIS include haemophilia A and B. ABRs are means (model-based or observed, as reported), with medians in parentheses where shown. Zero-bleed percentages refer to the periods indicated, value not presented in this table.

ABR, annualised bleeding rate (bleeds/year); ADA, anti-drug antibodies; ALT, alanine aminotransferase; aPCC, activated prothrombin complex concentrate; AT, antithrombin; FVIII, factor VIII; ISR, injection-site reaction; IV, intravenous; SC, subcutaneous; siRNA, small interfering RNA; TE, thromboembolic event; TFPI, tissue factor pathway inhibitor; TMA, thrombotic microangiopathy.

a ABR range across arms treated continuously for 52 weeks (26-week main phase plus 26-week extension).

b Additional thrombotic events unrelated to aPCC were reported.

c Dose and interval adjusted according to AT activity.

d Randomised concizumab arm; primary cutoff after ≥32 weeks, zero bleeds assessed over the first 24 weeks.

e Prior on-demand subgroup.

f Declining FVIII expression during follow-up; commercial withdrawal announced in February 2026 for reasons unrelated to efficacy or safety.

Two further observations sharpen the point. First, gene therapy, once presented as the definitive answer, illustrates how quickly a purely efficacy-driven narrative can unravel: valoctocogene roxaparvovec delivered durable bleed reduction over 5 years but with steadily waning and highly variable FVIII expression [12,13], and has now been voluntarily withdrawn from the market for commercial rather than safety reasons [14], while Pfizer discontinued development of giroctocogene fitelparvovec despite meeting its phase 3 endpoint [15]. Efficacy alone did not secure their place. Second, and more technically, when ABRs approach 0, the metric itself becomes statistically fragile: the wide confidence intervals the authors transparently acknowledge for the small Arm 1 extension subgroups (eg, 0.67 [95% CI, 0.13-3.61]) [1] are an inevitable consequence of modeling rare events in small samples, and they make head-to-head ranking between already-excellent therapies essentially impossible. We are approaching a floor effect against which the ABR can no longer discriminate.

## 3 Safety: Reassuring Here, but a Moving Target Across the Class

The safety profile reported for denecimig is reassuring and deserves emphasis. Over 52 weeks, there were no thromboembolic events, no hypersensitivity reactions, and no clinical evidence of neutralizing anti-denecimig antibodies; injection-site reactions occurred in <2% of injections and were uniformly mild and transient [1]. This is exactly the profile one hopes for, and it stands in welcome contrast to some of the cautionary tales that have shaped the field.

It is worth recalling why vigilance remains warranted. The early experience with emicizumab was marked by cases of thrombotic microangiopathy and thromboembolism, all associated with concomitant high-dose activated prothrombin complex concentrate (FEIBA) for breakthrough bleeding [16], a lesson that permanently reshaped bypassing-agent guidance. The rebalancing agents have written their own chapters: concizumab development was paused after nonfatal thromboembolic events before dose-capping restored an acceptable profile [9,10]; fitusiran’s history includes a fatal thrombotic event under its original fixed-dose regimen, mitigated only once dosing was tethered to a measurable antithrombin target of 15–35% [8]; and befovacimab was abandoned after CNS thromboses. The unifying theme is that these newer molecules act by raising the hemostatic ceiling, and that markers of thrombin generation rise dose-dependently even in patients who never thrombose, a reminder, echoed by the classical epidemiology linking high FVIII levels to venous thrombosis [17], that “more hemostasis” is not indefinitely “better hemostasis.” Against this backdrop, denecimig’s clean thrombotic record over a year is genuinely welcome, but it will need confirmation in larger, longer real-world exposure.

The second safety dimension that FRONTIER2 handles well, but which the field handles poorly, is immunogenicity. The absence of neutralizing anti-denecimig antibodies is reassuring, yet the experience with other bispecifics shows how consequential and difficult to characterize antidrug antibodies (ADAs) can be. Across the phase 3 emicizumab program, ADAs developed in about 5% of patients, roughly half neutralizing in vitro, but clinically relevant loss of efficacy remained rare [18]; the published neutralizing cases almost all presented within the first 25 weeks with unexpected bleeding and a prolonged aPTT [19]. Crucially, distinguishing a truly neutralizing antibody from a non-neutralizing one, or from simple nonadherence, still depends on nonstandardized, laboratory-developed assays [20]. The signal from the next-generation bispecific NXT007 is instructive: in the NXTAGE study, 73% of participants tested ADA-positive, yet only 2 of 30 showed any impact on drug exposure [21], a vivid illustration that raw ADA positivity is almost meaningless without a functional, neutralizing readout. The broader message for future trials, and for daily practice, is that we should invest now in validated assays measuring drug concentration and neutralizing capacity, so suspected loss of efficacy can be worked up rationally rather than guessed at.

## 4 Quality of Life: Probably the Most Important Criterion we Still Measure Worst

If the ABR can no longer separate excellent therapies, patient-reported outcomes and quality of life may be where the real differences exist. FRONTIER2 is commendable here, reporting reduced treatment burden (a mean Hemo-TEM reduction of up to 16 points, well beyond the 8-point clinically meaningful threshold), improved physical functioning and reduced joint pain [1]. These are the outcomes that patients increasingly tell us matter the most—the shift from merely preventing bleeds toward what Krumb and Hermans have called a “hemophilia-free mind”; freedom not only from bleeding but from the permanent mental burden of the disease [22,23].

Two caveats and 1 opportunity follow. The caveat, which the authors and Reviewer both rightly flag, is methodological: the extension phase is uncontrolled, and the Haemophilia Patient Preference Questionnaire used to capture the “strong preference” for denecimig is bespoke and not yet validated [1]. Preference and treatment-burden data are among the most visible positive messages of any trial, and they deserve validated instruments; the Hemo-TEM itself is a good example of the rigorous psychometric development the field should demand [24]. The opportunity lies in the direction of travel: patients now aspire to the least intrusive treatment compatible with protection. Subcutaneous administration, a prefilled pen rather than a vial, a fixed low injection volume, and above all extended dosing intervals are no longer conveniences but genuine differentiators—denecimig’s once-monthly option is a real advance. The competitive pressure on this axis is intense: NXT007 is being developed for monthly subcutaneous dosing, and, most strikingly, Novo Nordisk’s Inno8, a VHH-based FVIIIa mimetic, is being explored via the oral route in early-phase trials [25]—the prospect of an oral therapy would redefine “treatment burden” entirely. Future comparative studies should also address the real-life questions trials rarely capture: participation in higher-risk sport, the perioperative management of patients on nonfactor prophylaxis, and the laboratory monitoring required when FVIII is coadministered, all of which weigh heavily on how “free” a patient truly feels.

## 5 The Missing Piece: Objective Joint Assessment and The Case for Imaging Endpoints

If I were to identify a single element whose absence most limits the FRONTIER2 extension and, in truth, most contemporary hemophilia trials, it would be objective, imaging-based joint assessment. The study reports resolution of clinically defined target joints and improvements in self-reported joint pain [1], which is valuable, but joint outcomes remain anchored to bleeding history and patient report rather than to what is actually happening inside the joint.

This matters because the “zero-bleed” patient is not necessarily the “zero-synovitis” patient. In a Dutch cohort of persons with severe hemophilia on lifelong prophylaxis, MRI revealed signs of previous subclinical bleeding, hemosiderin deposition, and sometimes synovial hypertrophy in 16% of joints with no history of clinical bleeds [26]. Point-of-care ultrasound similarly detects hypertrophic synovium and osteochondral damage in patients whose clinical joint scores are near-normal [27]. In most patients on emicizumab prophylaxis, no laboratory parameter predicted spontaneous joint bleeding; however, ultrasound-detected synovitis and a higher HEAD-US score did predict spontaneous joint bleeding [28]. Validated, reproducible tools already exist for this purpose—the HEAD-US ultrasound protocol [29] and the IPSG additive MRI scale; they are precisely what is needed to move beyond the subjectivity of self-reported bleeds that the authors themselves acknowledge as a limitation, and which is compounded when patients previously on on-demand treatment differ in how they perceive and report bleeds [1].

The implication is straightforward. For a class of therapies that now routinely drives the ABR toward zero, preserving joint health, the outcome that actually determines lifelong disability is the endpoint that matters most and the one we measure least. Incorporating structured ultrasound or MRI endpoints into future clinical trials would not only provide a far more sensitive measure of true efficacy but would also give the field a way to discriminate between therapies that all look identical on the ABR. I would go further: a composite “zero bleed, zero synovitis” target, underpinned by imaging, may be the yardstick this crowded field needs.

## 6 Conclusion

Denecimig, on the evidence of the FRONTIER2 extension, is an excellent molecule: highly effective through a full year and across inhibitor status, reassuringly safe over the period studied, well tolerated, and offering a genuinely attractive low-burden administration profile including once-monthly dosing [1]. But its very quality illustrates the central paradox of contemporary hemophilia care: when almost every new therapy succeeds by the classical measure, that measure can no longer tell us which to choose. The therapeutic pipeline is beginning, quite seriously, to congest. If innovation in hemophilia A is to continue finding its place, and if patients are to benefit from that innovation rather than merely be dazzled by it, we will need to enrich the criteria by which we judge it: more granular, long-term safety, and immunogenicity data; validated patient-reported and preference instruments; and most urgently, objective imaging of joint health. Many are called; deciding who is chosen will require better tools than the ones that got us here.
